# Transcriptome Analysis of Salt Tolerant Common Bean (*Phaseolus vulgaris* L.) under Saline Conditions

**DOI:** 10.1371/journal.pone.0092598

**Published:** 2014-03-20

**Authors:** Mahmut Can Hiz, Balkan Canher, Harun Niron, Muge Turet

**Affiliations:** Bogazici University Department of Molecular Biology and Genetics, Istanbul, Turkey; USDA-ARS-SRRC, United States of America

## Abstract

Salinity is one of the important abiotic stress factors that limit crop production. Common bean, *Phaseolus vulgaris* L., a major protein source in developing countries, is highly affected by soil salinity and the information on genes that play a role in salt tolerance is scarce. We aimed to identify differentially expressed genes (DEGs) and related pathways by comprehensive analysis of transcriptomes of both root and leaf tissues of the tolerant genotype grown under saline and control conditions in hydroponic system. We have generated a total of 158 million high-quality reads which were assembled into 83,774 all-unigenes with a mean length of 813 bp and N50 of 1,449 bp. Among the all-unigenes, 58,171 were assigned with Nr annotations after homology analyses. It was revealed that 6,422 and 4,555 all-unigenes were differentially expressed upon salt stress in leaf and root tissues respectively. Validation of the RNA-seq quantifications (RPKM values) was performed by qRT-PCR (Quantitative Reverse Transcription PCR) analyses. Enrichment analyses of DEGs based on GO and KEGG databases have shown that both leaf and root tissues regulate energy metabolism, transmembrane transport activity, and secondary metabolites to cope with salinity. A total of 2,678 putative common bean transcription factors were identified and classified under 59 transcription factor families; among them 441 were salt responsive. The data generated in this study will help in understanding the fundamentals of salt tolerance in common bean and will provide resources for functional genomic studies.

## Introduction

Soil salinity is one of the most severe abiotic stress factors limiting the productivity of agriculture. Although most plants are glycophytes that are highly sensitive to saline environment, halophytes are plants that naturally grow under saline conditions throughout their life cycle. Salinity effects nearly 20% of all irrigated lands worldwide [1] and expected to reach around 50% in the near future [2]. A soil is considered saline if the electrical conductivity of its saturation (EC) is above 4 dS/m [3] which is equivalent to approximately 40 mM NaCl.

As a member of grain legumes and a glycophyte crop, common bean (*Phaseolus vulgaris* L.) is a major source of human dietary protein, minerals, vitamins, and represents nearly half of the consumed grain legumes worldwide [4]. Common bean is also vital in agriculture as it forms root nodules via symbiotic associations with nitrogen fixing bacteria [4]. Nearly 60% (360 Mt) of the annual fresh bean production in Turkey (FAO:http://faostat.fao.org/faostat) is highly dominated in Black Sea region where the soil salinity levels can reach up to 2–4 dS/m [5]. However, it is known that even at 1 dS/m salinity level, the productivity of common bean can be reduced up to 20% [6].Thus, understanding the fundamentals of salt tolerance in common bean, eventual development of improved varieties and their introduction to saline environments are imperative in agriculture.

Although halophytes may use avoidance mechanisms, glycophytes tolerate salinity by minimizing ion disequilibrium and the consequent secondary effects [7]. In other words, tolerance mechanisms require concerted actions of mechano-receptors, ion transport channels, and secondary signal molecules to maintain ion homeostasis as well as cascades of gene activations for hormonal metabolism, signal transduction pathways, and stress responses [8]–[12].

Considering the multifactorial nature of tolerance responses, development of tolerant plants for the benefit of sustainable crop improvement still awaits accumulation of additional knowledge about the identity of components that are involved in this process.

Recent developments in high-throughput approaches to study gene expression profile have emerged as an important tool to understand how plants respond to biotic and abiotic stresses. In the last few years, there have been accumulating reports on RNA-sequencing data and expression profiling on both model plants and agriculturally important crops [13]–[21] to identify genes involved in stress responses. Although, recently such high throughput transcriptome assemblies have been started in legumes [22]–[30], there are still only a handful of studies regarding the transcriptome analysis under abiotic stress conditions in these species. These studies include the effects of drought, saline-alkaline conditions and salt stress in gene expression profiling of chickpea [31], soybean [32], *Medicago truncatula* [33], and alfa alfa [21] respectively. However, there is no such transcriptome analysis under abiotic stress available yet for common bean. In combination with continuously generated reference genome sequences for diverse legume species [34]–[39], next generation RNA sequence analyses will provide valuable information for both identification and cloning of stress tolerance genes which can be used to improve varieties with enhanced tolerance mechanisms.

In this study, we used the Illumina high-throughput RNA-sequencing platform for transcriptomic analysis of a salt tolerant common bean, Ispir genotype. We aimed to identify differentially expressed genes (DEGs) and related pathways by comprehensive analysis of data from both root and leaf tissues of the tolerant genotype grown under saline and control conditions in a hydroponic system. *De novo* assemblies of the sequencing data, functional annotations of unigenes, and their characterization with gene ontology and metabolic pathway analysis provided potential lists of candidate genes. Functional identification of these candidates using reverse genetics approaches in our ongoing studies will contribute to the understanding of salt tolerance mechanisms.

## Methods

### Plant growth and salt treatments

The seeds of salt tolerant “Ispir” variety were kindly supplied by Prof. H. Yildiz Dasgan (Cukurova University Department of Horticulture, Adana, Turkey). The seeds were surface sterilized in a solution containing 5% (v/v) hypochlorite for 5 min and rinsed three times with distilled water. The seeds were germinated in vermiculite containing plug trays at 24/20°C cycle under a 16-h light/8-h dark photoperiod with 300 μmol m^−2^ s^−1^ light intensity, and 50–60% relative humidity up to the fully expanded foliage stage by daily watering with hydroponic nutrient solution containing 3 mM Ca(NO_3_)_2_, 1 mM MgSO_4_, 0.9 mM K_2_SO_4_, 0.2 mM KH_2_PO_4_, 0.1 mM FeEDTA, 10 nM H_3_BO_3_, 1 nM MnSO_4_, 1 nM ZnSO_4_, 0.1 nM CuSO_4_, and 0.01 nM (NH)_6_Mo_7_O_24_ [40]. Eight seedlings were transferred to two pots (four seedlings each) containing hydroponic nutrient solution that was replenished daily. During salt treatment, the photoperiod was also kept the same as in the germination conditions. Once plants reached trifoliate stage (five days post transfer), one pot was left as control and the other was exposed to gradually increasing NaCl concentrations. In this study, the hydroponics system was preferred to keep the nutrients and NaCl levels under strict control to achieve homogenous growth of the plants. Furthermore to minimize the risk of plasmolysis due to the osmotic shock [41] during salt treatments “gradual step acclimation” method was used [42], thus salt application was started with 50 mM in the first day, followed by 100 mM in the second day, and reached to 125 mM between days three to five in hydroponic nutrient solution.

### Sample collection and RNA isolation

The root and the leaf tissues from salt treated and control plants were sampled at the fifth day of the salt treatment. Samples were frozen in liquid nitrogen and stored at −80°C prior to RNA extractions. Total RNAs were extracted with RNeasy Plant kit (Qiagen, Hilden, Germany) according to the manufacturer's instructions. The concentrations of RNA samples were determined by NanoDrop 1000 spectrophotometer (Thermo Fisher Scientific, Wilmington, DE). RNA quality was assessed by 1% denaturing agarose gel electrophoresis.

**Figure 1:**
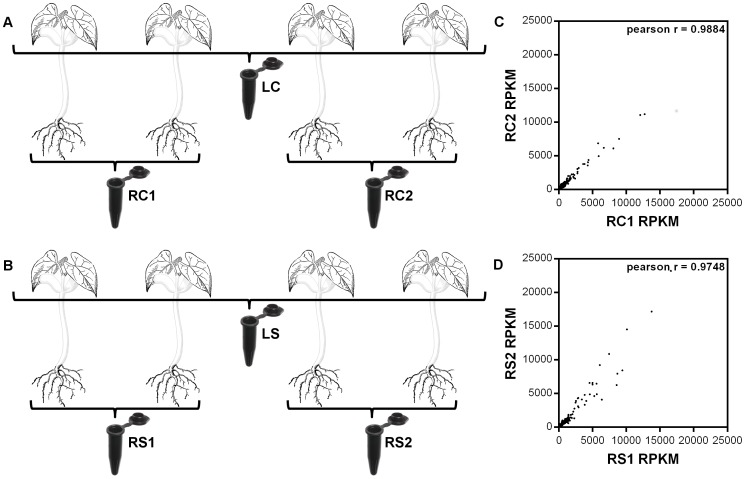
Schematic representation of the sample pooling strategy and correlation analysis of root samples RPKM values. Black lines indicate the pooling of leaf and root samples from control plants (A) and salt treated plants (B). Graphs show the correlation analysis within root control (C) and salt treated (D) samples. Nomenclature: LC: Leaf Control; LS: Leaf Salt treated; RC1 and RC2: Root Control 1 and 2; RS1 and RS2: Root Salt treated 1 and 2.

Initially, RNA samples from root tissues of two control (RC: Root Control; RC1 and RC2) as well as two salt treated plants (RS: Root-Salt treated; RS1 and RS2) were isolated as two biological replicates, and they were pooled as described in Figure 1A and 1B. Forty μg of total RNA from the two biological replicates were sequenced by Illumina HiSeqTM 2000 system (BGI, Shenzen, China). Pearson correlation coefficients between the RPKM (reads per kilobase per million reads) values of the two biological replicates were calculated as 0.99 and 0.97 for control and treated samples respectively (Figure 1C and 1D). Due to the observation of high correlation within the biological replicates of root tissues, we pooled RNA samples directly from leaf tissues of the same four plants from both control (LC: Leaf Control) and treated samples as a cost effective strategy [43], [44].

### cDNA library construction and Illumina sequencing

RNA quality and quantity were verified using Nanodrop 1000 spectrophotometer and Agilent 2100 bioanalyzer before cDNA library generation at BGI (Shenzen, China). Total RNAs were treated with DNase I before poly (A+) mRNA enrichment with oligo dT magnetic beads. Poly (A+) RNAs were digested into 200–700 nt fragments by RNA Fragmentation Reagent, and random hexamer primed poly (A+) RNA fragments were transcribed into first-strand cDNAs. Subsequently in the presence of dNTPs, RNase H, and DNA polymerase I, the second strands were synthesized, purified using QiaQuick PCR purification kit (Qiagen, Hilden, Germany), and used for end repair single adenine nucleotide addition. The sequencing adaptors were ligated to the fragments. The paired end library constructions were finalized by size selection with agarose gel electrophoresis and selective enrichment of the cDNA fragments with PCR amplification. The libraries were sequenced on a flow cell using Illumina HiSeq2000 sequencing instrument after quality control with Agilent 2100 bioanalyzer and qPCR to detect fragment size and concentration.

### *De novo* assembly and data analyses

Subsequent to adapter trimming, the raw data were filtered for reads with more than 5% ambigous bases and/or low quality reads with bases 20% of which has a Phred score less than 10. The clean reads from each library were *de novo* assembled into contigs with Trinity software [45] (http://sourceforge.net/projects/trinityrnaseq/files) setting k-mer length to 25. This has generated a total of six subtranscriptomes. Contigs of the subtranscriptomes were pooled, clustered, and assembled using the Trinity software to obtain sequences that can no longer be extended on either end, and they were referred as all-unigenes. All-unigene sequences were aligned using BLASTx against NCBI non-redundant (Nr) protein, Swiss-Prot protein, the Kyoto Encyclopedia of Genes and Genomes (KEGG) pathway, and Cluster of Orthologous Groups (COG) databases to determine sequence orientations and protein coding region predictions. Proteins with the highest ranks in BLASTx alignment results were used to predict coding region sequences. All-unigenes that could not be aligned to sequences in any of the databases mentioned above were scanned by ESTScan software [46] to predict sequence orientations and coding regions. For annotations, all-unigenes were searched against the Nr database using BLASTx with 10^−5^ as *E*-value cut-off point and sequences with the highest similarities were retrieved. To obtain Gene Ontology (GO) terms regarding biological process, molecular function and cellular component [47] descriptions, the resulting BLASTx hits were analyzed by Blast2GO software [48]. The GO annotations were functionally classified by WEGO [49] software for gene function distributions of common bean species at macro level. BLASTx analysis against the KEGG pathway database was also performed to assign putative metabolic pathways to all-unigenes.

To estimate gene expression levels, six (four from the root libraries, two from the leaf libraries) RPKM values were calculated for every all-unigene by mapping six subtranscriptomes with SOAP2 software [50], applying three mismatches as threshold. The clean reads mapped to more than one all-unigene were not used to calculate RPKM. Corrections for false positive and false negative errors were performed by calculating the FDR (false discovery rate) values [51]. The DEGs were selected by using a FDR-value ≤0.001 and the absolute ratio of log_2_ (RPKM-tr/RPKM-cont) ≥1 as threshold values. The GO terms and the KEGG pathways that were enriched within the DEGs were identified by publicly available agriGO [52] and FatiGO [53] software respectively. To analyze the functional significance of enriched terms hypergeometric tests were employed by using the common bean transcriptome as background, setting the FDR and the Adjusted *P*-values lower than 0.05 for the agriGO and FatiGO software respectively.

### qRT-PCR analyses

Quantitative Reverse Transcription PCR (qRT-PCR) analyses were performed for 43 unigenes (Table S1) using the *insulin degrading enzyme* (*IDE*, Unigene29213) [GenBank: FE702602.1] and the *actin-11* (*Act-11*, CL442.Contig3) [GenBank: CV529679.1] genes of common bean as stably expressed internal references under salt stress [54]. Four individual plants from both control and treated groups were used in qRT-PCR analyses as biological replicates. For each biological replicates, three qRT-PCR reactions were performed as technical replicates. Single stranded cDNAs were synthesized from one μg of total RNA using RevertAid First Strand cDNA Synthesis Kit (Thermo Fisher Scientific, Wilmington, DE). Gene-specific primers were designed using the Primer Design module of CLC Main Workbench (version 6.0) software. qRT-PCR analyses were performed by PikoReal 96 Real-time PCR system (Thermo Fisher Scientific, Wilmington, DE) using 10 ng first strand cDNAs and AccuPower 2X Greenstar master mix (Bioneer, Daejon, Korea) with 0.25 pmol forward and reverse primers in a 10 μl solution. Upon initial denaturation at 95°C for five min., 40 cycles of denaturation at 95°C for 10 s and 60 s of annealing, and extension steps were performed at specific temperatures (Table S2) optimized for each primer pair. The relative expression levels were calculated using the 2^–ΔΔCt^ method for each gene and were normalized to the geometric average of Ct (threshold cycle) values of the internal reference genes. The Student's *t*-test was applied to determine the significant differences in expression levels between treated and control samples collected at the fifth day of salt treatment.

### Identification of putative transcription factors

Sequences for Hidden Markov Model (HMM) motifs of the transcription factors (TFs) belonging to *Glycine max*, *Medicago truncatula* and *Lotus japonicus* were acquired from the Legume Transcription Factor Database (Legume TFDB) [55], [56] and combined to create a single legume motif database consisting of 61 TF families. An all-unigene was accepted as a putative TF if it has shown 90% or more sequence homology (*E*-value ≤10^−10^) with any of the HMM motifs of at least one of the three legume species. The putative TFs within the all-unigenes and the DEGs were categorized based on the TF families and their presence in root or leaf tissues.

## Results

### *De novo* assembly of the sequencing data

Previous reports have emphasized that during NaCl applications, Na^+^ concentrations reach to toxic levels and plants start to react to salt stress rather than osmotic shock only after 24–72 hours gradual step acclimation [41], [42]. In our studies with NaCl treatment on Ispir variety have also shown that detectable physiological effects of salt stress become significant at the fifth day (72 hours after gradual step acclimation) of treatment in hydroponics cultures (data not shown). Therefore, the sequencing was performed on the leaf and the root RNA samples collected at this time point. The Illumina sequencing was performed separately for each RNA sample (LC, LS, RC1, RC2, RS1, and RS2) and six subtranscriptomes (Figure 1A and 1B) were generated with 90-bp raw reads. More than 158 million clean reads remained from the six subtranscriptomes (Table 1) with ∼97% Q20 bases (i.e. percentage of sequences with sequencing error rate lower than 1%) that constituted over 14 GBase (7.05×10^9^ nt in control, and 7.23×10^9^ nt in salt treated samples) of data after quality assessment and data filtering. All clean reads were deposited in the NCBI Short Read Archive (SRA) database and can be accessed with SRP029243 accession number.

**Table 1:** Statistics of raw data for *P.vulgaris* L. transcriptome.

Samples	Total Reads	Total Nucleotides (nt)	Q20 percentage	GC percentage
1LC	25 907 332	2 331 659 880	97.02%	46.02%
2RC	26 481 724	2 383 355 160	96.91%	46.70%
3RC	25 998 386	2 339 854 740	96.94%	46.97%
1LS	27 191 626	2 447 246 340	97.12%	46.19%
2RS	27 280 462	2 455 241 580	96.91%	47.08%
3RS	25 821 046	2 323 894 140	96.83%	47.30%

The clean reads from LC, LS, RC1, RC2, RS1, and RS2 subtranscriptomes were *de novo* assembled using the Trinity software, which generated 52,858, 51,564, 60,590, 64,986, 59,510, and 58,174 unigenes, respectively (Table 2). Further assembly of the subtranscriptomes generated the common bean transcriptome consisting of 83,774 all-unigenes. The total length of all-unigenes was 68,147,816 bp with a mean length of 813 bp and N50 of 1,449 bp (i.e 50% of the assembled bases were incorporated into all-unigenes of 1,449 bp or longer) (Table 2). The lengths of all-unigenes ranged between 150 and 17,022 bp. In total, 55,471 (66.72%) all-unigenes were unique and the remaining 28,303 (33.8%) fell into 5,403 clusters in which the number of unigenes ranged between 2 and 42. Among the 83,774 all-unigenes, 63,011 (71% Nr-annotated) were expressed in the leaf samples whereas 73,523 (69% Nr-annotated) were expressed in the root samples. Randomly distributed clean reads from the six subtranscriptomes evenly covered the common bean transcriptome with an average of ∼20 fold coverage depth (Figure S1).

**Table 2:** Statistics analysis for unigene assembly of *P. vulgaris L*.

Length of Unigenes (bp)	Number of unigenes						
	LC	RC1	RC2	LS	RS1	RS2	All
150–500	31659	37290	41636	29684	38096	37683	44558
500–1000	11135	12780	12836	10819	12194	11878	15702
1000–1500	5104	5499	5514	5280	4939	4612	9754
1500–2000	2622	2675	2678	2972	2352	2202	6115
≥ 2000	2338	2346	2322	2809	1929	1799	7645
Total	52858	60590	64986	51564	59510	58174	83774
Mean length	644	613	585	685	579	568	813
N50 (bp)	1016	946	906	1121	866	843	1449
Total Length (bp)	34018974	37138056	38042659	35328894	34441515	33036044	68147816

### Annotation and classification of common bean transcriptome

The BLASTx searches (*E*-value ≤ 10^−5^) revealed that out of 83,774 all-unigenes, 58,171 (69.4%), 44,174 (52.7%), and 28,564 (34.1%) showed significant similarity to the sequences in Nr, Swiss-Prot and KEGG databases respectively. Among the all-unigenes, 43,519 (51.9%) were commonly annotated in Nr and Swiss-Prot; 41,087 (49%) in Nr and KEGG; 27,116 (32.4%) in KEGG and Swiss-Prot, and 27,078 (32.3%) were annotated in all three databases. For the 91% of the Nr -annotated sequences, the *E*-values were less than or equal to 10^−10^. In addition, 24,909 (29.7%) all-unigenes did not match significantly to any of the sequences in these databases.

**Figure 2:**
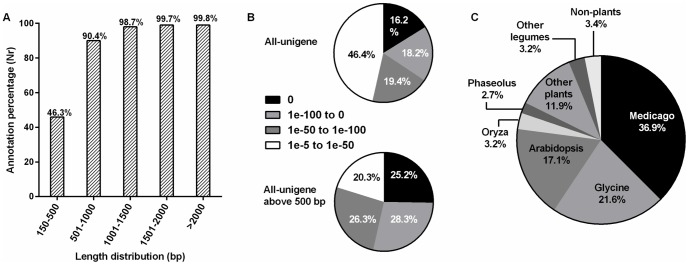
Reliability analysis of all-unigene annotations. Histogram of all-unigenes according to the Nr database annotations with 500 bp intervals (A). E-value distributions for Nr annotations based on BLASTx analysis; panel B1 represent the percentage distributions of all-unigenes and panel B2 represent all-unigenes longer than 500 bp. Genera distribution of all-unigenes according to the Nr database annotations (C).

More than 46% of all-unigenes smaller than 500 bp had BLASTx hits in the Nr database whereas for those longer than 500 bp the ratio was over 90.4% (Figure 2A). This has indicated that the longer the all-unigenes were the more they were likely to have Nr annotations and the lower *E*-values than the shorter all-unigenes (Figure 2B). The great majority (over 96%) of Nr annotated all-unigenes showed the highest homology to the plant proteins (Figure 2C). Furthermore, 64.4% were specifically annotated with sequences from legume species (Figure 2C), which indicated the reliability of our transcriptome analysis. In our data set, we also found few all-unigenes, which were annotated by phytoplankton and plant pathogens proteins, most probably due to the hydroponic growth conditions used in this study. Similar to the observation in an earlier report [57], 96 all-unigenes expressed exclusively in roots were Nr annotated from *Phytophthora sojae*, which is a soybean pathogen that causes rotting of stem and roots [58]. This observation indicates that our transcriptome analysis detected transcripts even in trace amounts.

Using WEGO software the 27,959 all-unigenes were assigned to a total of 98,948 GO annotations which fall into three main categories; 19,154 all-unigenes were within 28 terms of the “Biological Process”category, 18,175 all-unigenes were within 15 terms of the “Cellular Component” category, and 22,190 all-unigenes were within 12 terms of the “Molecular Function” category (Figure S2).

All-unigenes were assigned to KEGG pathway and COG databases, a total of 28,564 all-unigenes were annotated in 125 individual pathways (Table S3) and a total of 23,141 all-unigenes were assigned to 25 functional classes respectively (Figure S3).

### qRT-PCR verification of RPKM based gene expression

**Figure 3:**
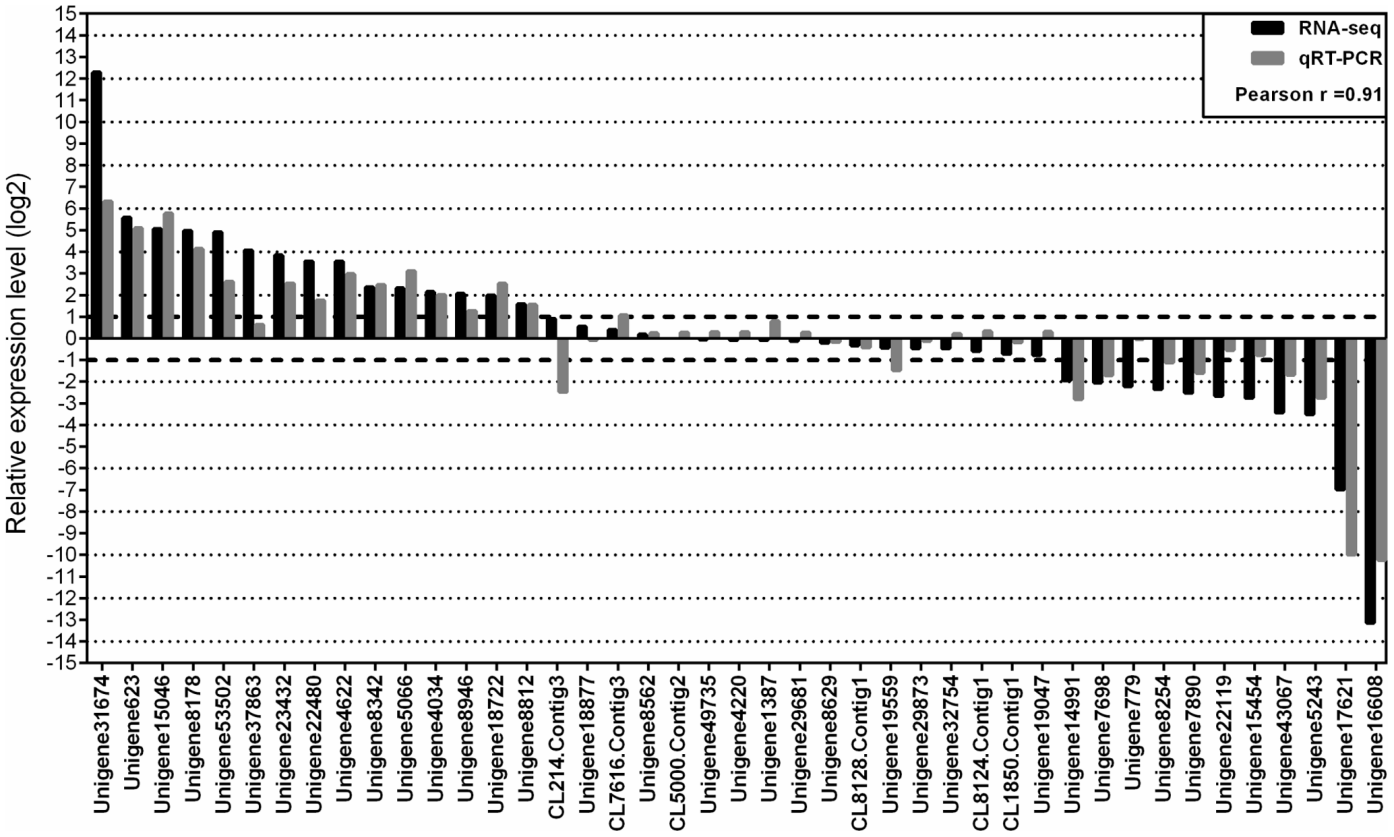
Relative expression level comparisons of qRT-PCR and RNA-seq results for selected all-unigenes. The qRTPCR results of 43 all-unigenes were compared to the RPKM values (log_2_ (RPKM-tr/RPKM-cont) ≥1).

We performed qRT-PCR analysis for 43 selected all-unigenes (Table S1) from root and leaf samples with specific primers (Table S2) to assess the reliability of our sequencing results (Figure 3). Among these selected all-unigenes, 15 were upregulated (log_2_ (RPKM tr/cont) ranged between 1.57 and 12.28), 17 were non-differentially expressed (log_2_ (RPKM tr/cont) ranged between −0.77 and 0.89), and 11 were downregulated (log_2_ (RPKM tr/cont) ranged between −1.18 and −13.13). The comparison of qRT-PCR and sequencing results revealed a high correlation for the selected unigenes (Pearson r = 0.91, Figure 3). In addition to high correlation between qRT-PCR and RNA-seq results, the production of expected fragment sizes using designed primers have also confirmed the reliability of *de novo* assembly.

### Identification and functional classification of DEGs

**Figure 4:**
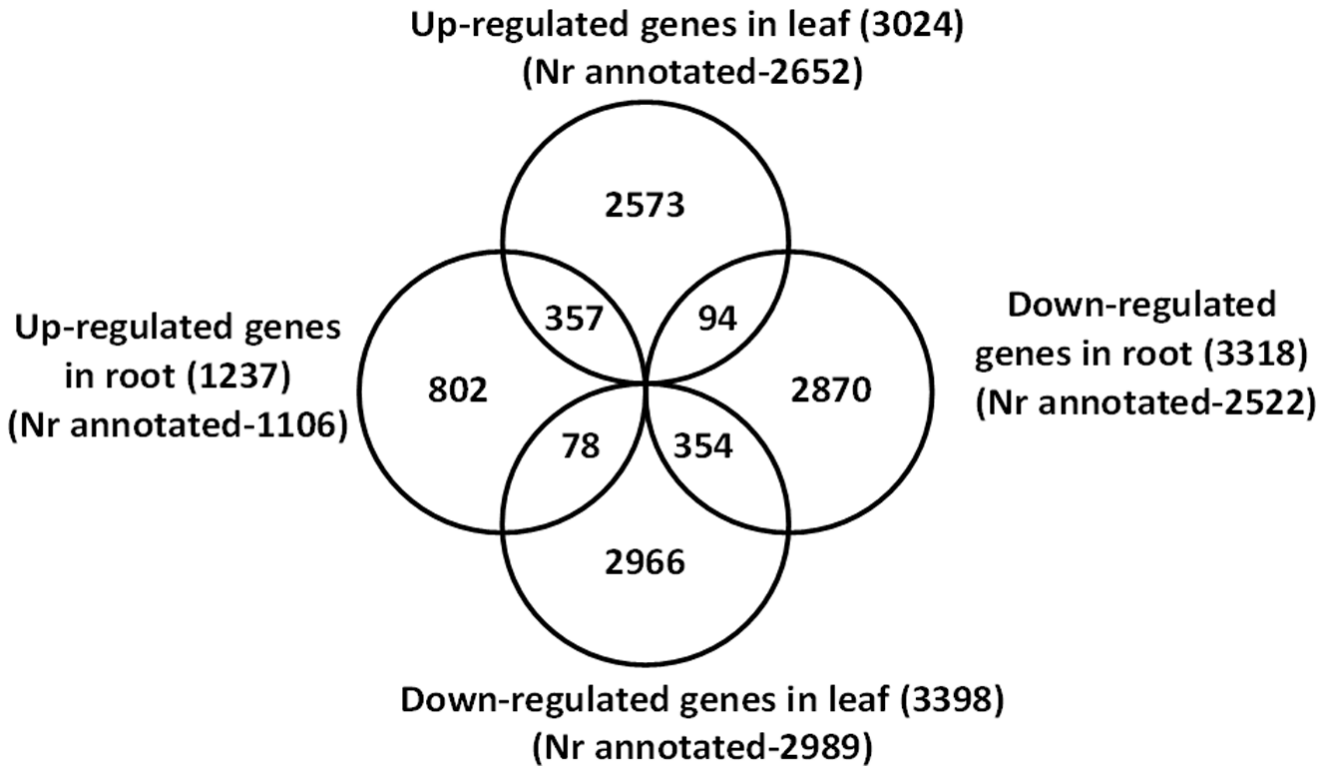
Venn diagram showing the number of DEGs. The sums of the numbers in each circle were indicated within the parantheses of the corresponding title.

Upon comparison against control groups, all-unigenes with more than or equal to two-fold change (│log_2_ (RPKM tr/cont) │≥1) in their gene expression level with a FDR value below 10^-3^ were defined as DEGs. Based on these criteria, the number of DEGs in leaves and roots were 6,422 and 4,555, respectively (Figure 4; Table S4 and S5). Out of 6,422 DEGs, 3,024 (88% Nr-annotated) were upregulated, and 3,398 (88% Nr-annotated) were downregulated in the leaf samples, whereas among the 4,555 DEGs of the roots, 1,237 (89% Nr-annotated) were upregulated, and 3,318 (76% Nr-annotated) were downregulated upon salt treatment (Figure 4).

**Figure 5:**
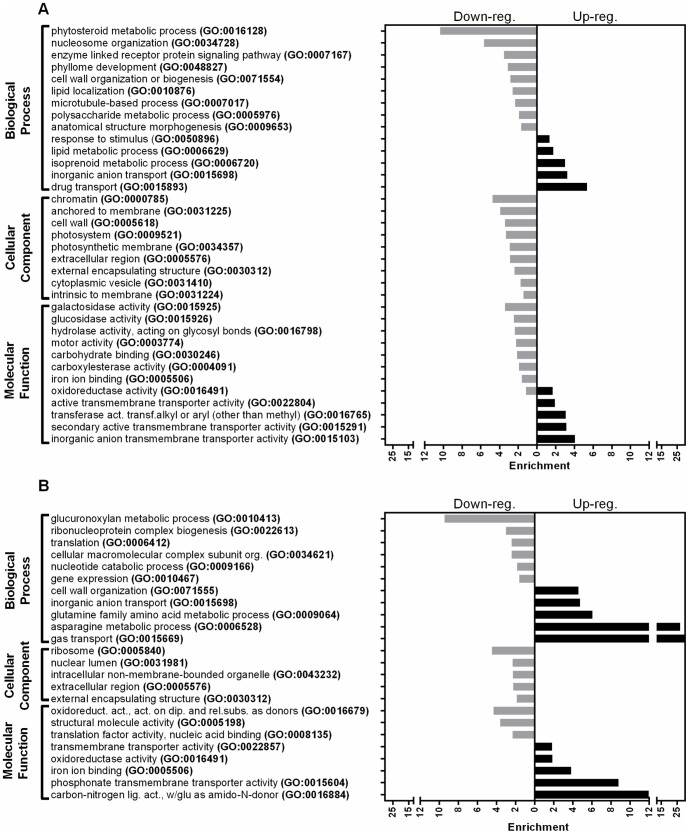
Gene Ontology functional enrichment analysis of DEGs. Panel A represents the DEGs in leaf tissues and panel B represents the DEGs in root tissues. (FDR-value <0.05).

**Figure 6:**
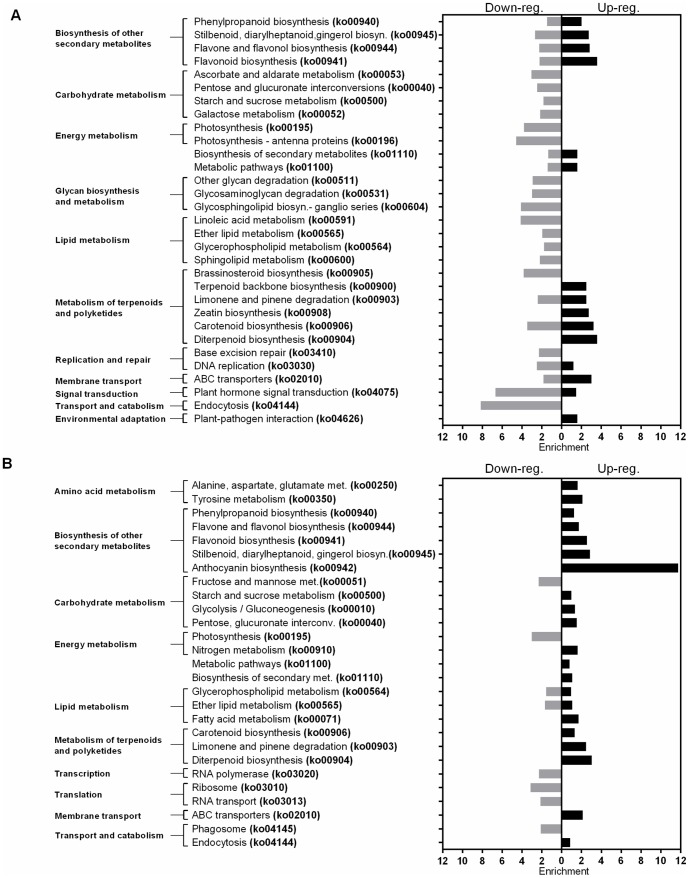
Pathway enrichment analysis of DEGs. Panel A represents the DEGs in leaf tissues and panel B represents the DEGs in root tissues. (Adjusted-*P*-value <0.05).

In both leaf and root tisssues, the enriched terms in the GO database (Figure 5, Table S6 and S7) were in agreement with the KEGG metabolic pathways (Figure 6, Table S8 and S9). Within the leaf tissues upregulated genes, the terms related with the secondary metabolite metabolism and the membrane transport activity were mostly enriched, however the macromolecular energy metabolism related terms were highly enriched within the downregulated genes (Figure 5A and 6A). Although, the DEGs in the root tissues have also shown a similar pattern with the leaf tissues for the terms related with the secondary metabolite metabolism and the membrane transport activity, the terms related with the macromolecular energy metabolism were only slightly enriched within the upregulated genes. Additionally, in root tissues, the transcription/translation related terms were notably enriched within the downregulated genes (Figure 5B and 6B).

### Identification of putative transcription factors (TFs)

In this study, 2,678 putative TFs were identified based on the homology analysis of the all-unigenes against a combined legume database which was constructed from *Glycine max*, *Medicago truncatula* and *Lotus japonicus* HMM motifs. The putative TFs were classified under 59 out of the 61 TF families present in this database. The most abundant 10 TF families were AP2_EREBP, bHLH, PHD, HB, (R1)R2R3_Myb, WRKY_Zn, NAC, bZIP, C3H-TypeI, and Myb_related (Table 3, Table S10). A total of 441(16%) TFs, 331 in the leaf, and 161 in the root tissues (Table 3 and Table S10) identified as salt responsive. The composition of most abundant TF families were preserved within the leaf DEGs except the C3H-Type I family (replaced by C2C2-Zn-CO like) however in the root DEGs, the bZIP, C3H-TypeI, and Myb_related HSF were replaced by the C2C2_Zn-Dof, and C2H2_Zn TF families (Table S10).

**Table 3:** Number of total and differentially expressed bean TFs on family basis.

	All-unigene			DEGs	
TFs	All	Leaf	Root	Leaf	Root
**AP2_EREBP**	222	163	173	42	25
**bHLH**	212	160	171	29	18
**PHD**	172	157	162	14	8
**HB**	157	115	112	18	7
**(R1)R2R3_Myb**	154	114	122	28	22
**WRKY_Zn**	144	117	122	20	14
**NAC**	122	95	104	20	13
**bZIP**	111	75	85	14	3
**C3H-TypeI**	97	90	91	7	3
**Myb_related**	94	79	78	16	3
**TOTAL**	2678	2141	2196	331	161

## Discussion

Common bean is an important grain legume that provides 30% of the protein intake in developing countries (FAO: http://faostat.fao.org/faostat), and the production suffers drastically even in slightly saline soils [6]. Presence of genetic diversity within the species toward salt tolerance [59] offers a valuable opportunity to identify the key elements that might play a role in salt tolerance. The common bean genotype “Ispir” used in this study is known to be a very old local variety named after Ispir county of Erzurum province where it has been produced by more than 150 years. The variety has been registered in 2008 and patented as “Geographic Trademark” by Turkish patent office (www.tpe.gov.tr). The ability of the variety to tolerate high salt concentrations even at germination stage without compromising from germination time was well documented [59].

There exists a substantial amount of reports regarding the physiological responses to salt stress in common bean [59], [60]. Also there is a considerable collection of ESTs from cDNA libraries obtained from different organs and root nodules of legumes [61], [62] including sequences related to abiotic stresses such as phosphorus starvation, rust disease resistance, and drought stress. Within the last few years, there has been increasing number of studies on transcriptome analysis of legume species using high-throughput RNA sequencing approach, among those regarding the effects of salinity were recently reported on *Medicago* [21], [33], *Glycine* [32], *Cicer* [31], [63], and *Millettia* [57]. However, there are only three transcriptomic studies in the literature on common bean conducted by high-throughput RNA-sequencing approach, one of which is a general transcriptome [24]assembly, the other is a data-mining of host resistance gene like sequences [64] within the transcriptome and the third one is on the sulfur metabolism in developing seeds [65]. Therefore, we aimed to perform a large-scale comparative transcriptome analysis in two different tissues of a salt tolerant common bean variety under salt stress to identify key elements and their related functional pathways, which may play a critical role in stress tolerance responses.

In our study, the assembly of the transcriptome relied on the *de novo* method. The reliability and the sensitivity of our transcriptome were demonstrated by the high percentage of annotations (i.e. Nr-annotations, 69.4%), the ability to detect the trace amounts of transcripts from plant pathogens as well as phytoplankton contaminations (due to hydroponic system), and the correlation between the qRT-PCR results and the RPKM values. Functional annotation of common bean transcriptome also revealed that the highly represented terms in the GO (Figure S2), KEGG (Table S3), and COG (Figure S3) databases were also commonly represented in previous legume transcriptome studies [57], [66], [67]. Additionally the two previous common bean transcriptome studies [24], [65] generated comparable number of all-unigenes (94,623 and 77,448 all-unigenes) with our transcriptome assembly (83,774 all-unigenes), although they used pyrosequencing platform.

Comparative transcriptional level analysis of salt treated and control samples using RPKM values revealed that 10094 (12%) all-unigenes were salt responsive (Figure 4, Table S4 and S5). Among them 8457 (84%) were Nr-annotated. Although we used the same (or higher) stringent threshold values (│log_2_ (RPKM tr/cont) │≥1, FDR ≤0.001) with the previous studies in legumes [19], [21], [33], a higher number of salt responsive genes were identified in this study except the recently reported study in *Milletia pinnata* [57]. The reasons which may contribute to these disparities could be the differences in sensitivity and resolution between the hybridization [19] and sequencing based platforms as well as the differences between the assembly software. Moreover, the differences between salt tolerance characteristics of *Milletia pinnata*, a transitional species between halophytes and glycophytes [57], and common bean, a glycophyte [6] might be another reason. Additionally, other contributing factors might include the plant growth conditions, direct exposure of plants to high salt concentrations, and the differences in sample collection times. The previous transcriptome studies focused on earlier time points and sudden applications of elevated salt concentrations, whereas our transcriptome analysis was performed using the “gradual step acclimated” plant material and thus reflected the salt responses rather than osmotic shock responses [41].

During salt stress within plant, all the major processes such as photosynthesis, protein synthesis, energy and lipid metabolisms are affected [68].Therefore, in order to tolerate this stress, plants require to initiate protective and survival actions by balancing cellular ion concentrations and minimizing ion toxicity through Na^+^/H^+^, K^+^ and Cl^-^ transporter/antiporter activities to eliminate water and nutrient deficits, regulate osmotic potential changes via synthesis of osmoprotectant and osmoregulants such as sugars, amino acids, amines, and also minimize tissue damage through activation of scavenging pathways to eliminate antioxidant productions [1], [69], [70].

Based on the enrichment analysis of the DEGs, both root and leaf tissue genes were closely related with stress tolerance mechanisms. Major categories of the stress related functions were transmembrane transport activities (such as GO:0031224, GO:0015698, GO:0015103, GO:0015893, ko02010), carbohydrate metabolism (such as GO:0005976, GO:0015925, G0:15926, ko00040, ko00500, ko00052), lipid metabolism (such as GO:0010876, GO:006629, ko00591, ko00565, ko00600), secondary metabolite metabolism and oxidoreductase activity (such as GO:0006720, GO:0016884, GO:0006528 GO:16491 GO:0016679, ko00900, ko00940, ko00944), and transcriptional/translational activities (such as GO:0034728, GO:0022613, GO:0006412, GO:0010467,ko03020, ko03010, ko03013) (Figure 5 and 6, Table S6, S7, S8, and S9).

Ionic imbalance causes ion toxicity due to replacement of K^+^ by Na^+^ ions via competitive transport of Na^+^ through potassium channels [71]. Although cytosolic K^+^ is essential as cofactor for several enzyme activities, Na^+^ has toxic effects and thus has to be excluded or sequestrated into vacuoles in the cells [71]. Therefore to achieve a high K^+^/Na^+^ ratio for improved salt tolerance, increased expression of Na^+^/H^+^ antiporters with driving force of vacuolar H^+^-ATPases and H^+^ pyrophosphatases are important. Importance of these antiporter activities was also implicated once more in our study as the leaf tissues have shown distinctive upregulation in Na^+^/H^+^ antiporter (CL4908.Contig3_All) as well as vacuolar H^+^ATPase genes (CL3536.Contig4_All). Additionally, upregulation in the nonselective cyclic nucleotide gated cation channels, *CNGC2* (CL5050.Contig1_All) in root tissues were striking in our salt tolerant variety, considering the previously implicated role of the CNGC channels in the main pathway of Na^+^ entry to roots [72]. However, studies in Arabidopsis has shown that when the K^+^ level was limited due to elevated Na^+^ during stress, activation of nonselective channel *At*CNGC family members were still crucial to increase K^+^ influx to the root cells even at the expense of Na^+^ influx [73], [74]. More strikingly, the *CNGC2* (CL5050.Contig1_All) was shown to be the only member that preferentially conducts K^+^ without the transport of Na^+^ [75].

Similar to Na^+^, Cl^−^ is also toxic to the cells, thus Cl^−^ homeostasis by endosomal compartmentalization is critical for plant adaptations to salt stress [76]. Although Cl^−^ transport genes have been poorly studied, the attention mainly focused on voltage-dependent Cl^−^ channels (Cl^−^/H^+^ antiporters) localized in endosomal membranes which may function in vacuolar sequestration of Cl^−^ to minimize the tissue necrosis and long distance transport in shoots as well as Cl^−^ exclusion from roots [77], [78]. Our enrichment analyses have also revealed upregulation of Cl^−^ channels and Cl^−^/NO_3_^−^ transporter genes in the leaf (CL5256.Contig5_All) and the root tissues (CL5256.Contig6_All) respectively. The actions of Cl^−^ channels were correlated with NO_3_^−^, another macronutrient univalent anion in plants, and Cl^−^/NO_3_^−^ interactions show analogy to Na^+^/K^+^ interactions and selectivity to Na^+^ exclusion [78] during salt tolerance.

Osmotic potential change in plants is a typical outcome of ionic imbalance during salt stress, which creates domino effect in the activation of multiple metabolic processes. One of these metabolic processes includes increase in the accumulation of highly hydrophilic organic compounds (osmolytes) and hydrophylic proteins [68]. Upregulation of both osmolyte biosynthesis enzymes (CL7986.Contig1_All, CL2135.Contig2_All, CL3755.Contig1_All, Unigene14850_All, CL6414.Contig1_All, Unigene27033_All) and the hydrophilic LEA proteins (CL3431.Contig2_All, Unigene12424_All) in both leaf and root transcriptomes were a good indication of their protective role of cytosolic components of cells during osmotic imbalance. Activation of metabolic processes also enhances demands on energy resources, which are supplied by cellular respirations [79]. Increases in the expression of the genes related with respiration pathway enzymes in both tissues were indicative of such catabolic activities (CL8031.Contig1_All, Unigene25787_All, Unigene20031_All, and CL237.Contig7_All). Catabolic activities bring high risk of oxidative stress in plants which is associated with antioxidant production enzymes [80] to eliminate free oxygen radical accumulation in cells. Several different antioxidant biosynthesis related genes were upregulated within our transcriptome (CL1487.Contig1_All, Unigene1099_All, Unigene20329_All, and Unigene5947_All). During the battle against the osmotic as well as ionic stress in salinity, plant tolerance requires considerable efforts in maintenance of cell structure integrity by reinforcements or reorganizations in membranes, and cell wall components [81 and references therein]. It was not surprising to observe considerable amount of upregulation of genes regarding such activities (CL8348.Contig1_All, Unigene29739_All, Unigene20233_All, and CL7042.Contig1_All) in our transcriptome.

When we evaluated the common bean transcriptome under salt stress the results suggested that tolerance responses requires highly obvious efforts regarding ionic and osmotic homeostasis through transmembrane transport activities, mobilization and utilization of energy reserves for protection and preservation of structural integrity via metabolite biosynthesis while avoiding energy consumption for growth related transcriptional/translational activities (Figure 5 and 6).

Several transcription factors play important roles in translating stress signals into changes in gene expression. Based on the BLASTx analysis against the combined Legume TFDB, we have identified a total of 2,678 TF genes, in our common bean transcriptome (Table 3, and S10). In an earlier report by Kalavacharla *et al*. (2011), a similar number of TFs (2,516) were also identified.

Almost all of the most abundant TF families identified in our study within the DEGs (Table 3 and Table S10) have been well studied and implicated to play a role in both biotic and abiotic stress responses in plants [82 and references therein].

DREB genes are well studied members of AP2-ERF family, suggested to function in drought, salt, heat, and cold stresses by both ABA dependent or ABA-independent signaling pathways [83 and references therein]. Overexpression of DREB genes in diverse species of plants under various promoters have provided improved tolerance in abiotic stresses.

Although the biological function of most bHLH family members has not yet been studied in plants [84], increase in the bHLH transcription in tolerant wheat variety during salt stress, and regulation of drought tolerance response in Arabidopsis were elucidated by the bHLH regulated ABA signaling [84]. Increased focus on bHLH genes might shed light on its involvement in salinity stress as observed from overexpressions of bHLH92 in Arabidopsis [85], and *OrbHLH001* in wild rice phloem tissues [86] conferring improved salt tolerance.

After the first sequencing of NAC family gene RD26 [87] NAC domain has been characterized based on the consensus sequences from *NAM, CUC2, and ATAF1/2* proteins [88]. Since then differential expressions and involvement of several NAC genes in abiotic stress responses have been demonstrated [89].

As one of the largest TF families, MYB is involved in several physiological and biochemical processes during abiotic stress responses [90]. Among the members, *TaMYB33* TF, which shows high similarity to R2R3-MYB proteins in rice and maize, [91] has been indicated in salt stress tolerance via ROS detoxification and osmotic balance reconstruction in wheat [92].

The regulatory role of WRKY family was also demonstrated in high salinity responses, Arabidopsis *WRKY8* was predominantly expressed in roots and functions of it was consistent with the changes in Na^+^/K^+^ concentrations [86].

Several members of PHD finger protein family were observed to respond to abiotic stresses differentially. Especially soybean *PHD2* was shown to be uniquely expressed in tolerant variety and suspected to provide tolerance by diminishing the oxidative stress through regulation of downstream genes [93]. Functional relevance of PHD family as chromatin-mediated transcriptional mediator was suggested through their involvement in activation or repression of *ING1* [94], *Pf1* [95], *TIF1* [96], and *KAP1* [97] genes.

The Homeobox-zip (HB family) TF members were suggested as excellent candidates to generate stress responses in transgenic plants and cotton root development regulation under salt stress involved *GmHB1* gene expression [98], [99].

Certain members of C3H-type family TFs (*AtSZF1* and *AtSZF2*) were shown to be involved in salt responses transiently within the first few minutes of salt exposure [100], however in our study the C3H-type TFs were not abundant in salt responsive genes (Table 3), most probably due to the differences in sample collection time.

C2C2-Dof TFs are involved in the photosynthetic gene expression of plants and Dof2-domains play a role as tissue-specific repressors in PEP carboxylase promoters, which suggest their regulatory activities in photosynthesis [101]. The ERF-domain of C2H2-Zn TFs were also shown to be important transcriptional repressors in responses to abiotic stresses, a key role of *Zat7* protein in defense response during salinity was indicated in Arabidopsis [102].

A vast number of HSF family members of TFs were compiled from nine species; major aspects of functions implied their role in gene expression of chaperons for stability, localization of cellular components as well as in regulation of abiotic stress responses [103].

## Conclusion

Our comprehensive transcriptome analysis of a salinity tolerant common bean variety by Illumina sequencing is the first transcriptomic study on the response of common bean under salt stress. The identified all-unigenes have been observed to be involved in similar pathways as in previous reports of legumes according to the GO, KEGG and COG analyses. Substantial upregulation of transmembrane transport activities indicates the efforts of common bean to maintain ionic and osmotic homeostasis. To do so, energy consumption seems to be shifted from growth related transcriptional/translational activities towards maintaining current structural integrity through metabolite biosynthesis. Analysis of TFs among DEGs has implicated well studied TF families with known roles in abiotic and biotic stress as well as those that were not strongly associated with salinity stress previously, such as bHLH family. Although many of the DEGs identified has been annotated in publicly available databases, there were DEGs with dramatic expression differences that has not been annotated or implicated in abiotic stress responses, which are awaiting functional characterization. Overall, transcription profiling and identification of DEGs have provided valuable information for salinity tolerance mechanisms that is indispensable to maximize yield and utilization of arid lands.

## Supporting Information

Figure S1 **Random distribution of clean reads**. The x-axis describes the number of reads and the y-axis indicates the number of clean reads mapped to relative positions in unigenes for the six subtranscriptomes. The orientations of the genes are in 5′ to 3′direction and the gene lengths are normalized.
(TIF)

Figure S2 **GO annotations of all-unigenes.** The annotations were performed with Blast2GO software. The length of each bar indicated the percentage of all unigenes falls under each GO terms. The x-axis is in logarithmic scale.
(TIF)

Figure S3 **COG function prediction of all-unigenes.** The possible functions of all-unigenes were predicted by alignment to COG database. Each letter in the x-axis represented the COG categories listed on the right of the graph.
(TIFF)

Table S1 **List of selected all-unigenes for comparison of qRT-PCR and RNA-seq analysis.**
(XLSX)

Table S2 **List of primers to amplify the selected all-unigenes for qRT-PCR analysis.**
(XLSX)

Table S3 **KEGG pathway annotations for all-unigenes.**
(XLSX)

Table S4 **Differentially expressed unigenes in leaf tissues.**
(XLSX)

Table S5 **Differentially expressed unigenes in root tissues.**
(XLSX)

Table S6 **Gene Ontology funtional enrichment analysis of leaf DEGs.** (FDR-value <0.05).
(XLSX)

Table S7 **Gene Ontology functional enrichment analysis of root DEGs.** (FDR-value <0.05).
(XLSX)

Table S8 **Pathway enrichment analysis of leaf DEGs.** (Adjusted-*P*-value <0.05).
(XLSX)

Table S9 **Pathway enrichment analysis of root DEGs.** (Adjusted-*P*-value <0.05).
(XLSX)

Table S10 **The number of genes in TF families.**
(XLSX)
