# Supplementary figures and images for: Transcriptome Analysis of Salt Tolerant Common Bean (Phaseolus vulgaris L.) under Saline Conditions
Source: PLoS One. 2014 Mar 20;9(3):e92598. doi: 10.1371/journal.pone.0092598 (PMC3961409; doi:10.1371/journal.pone.0092598)

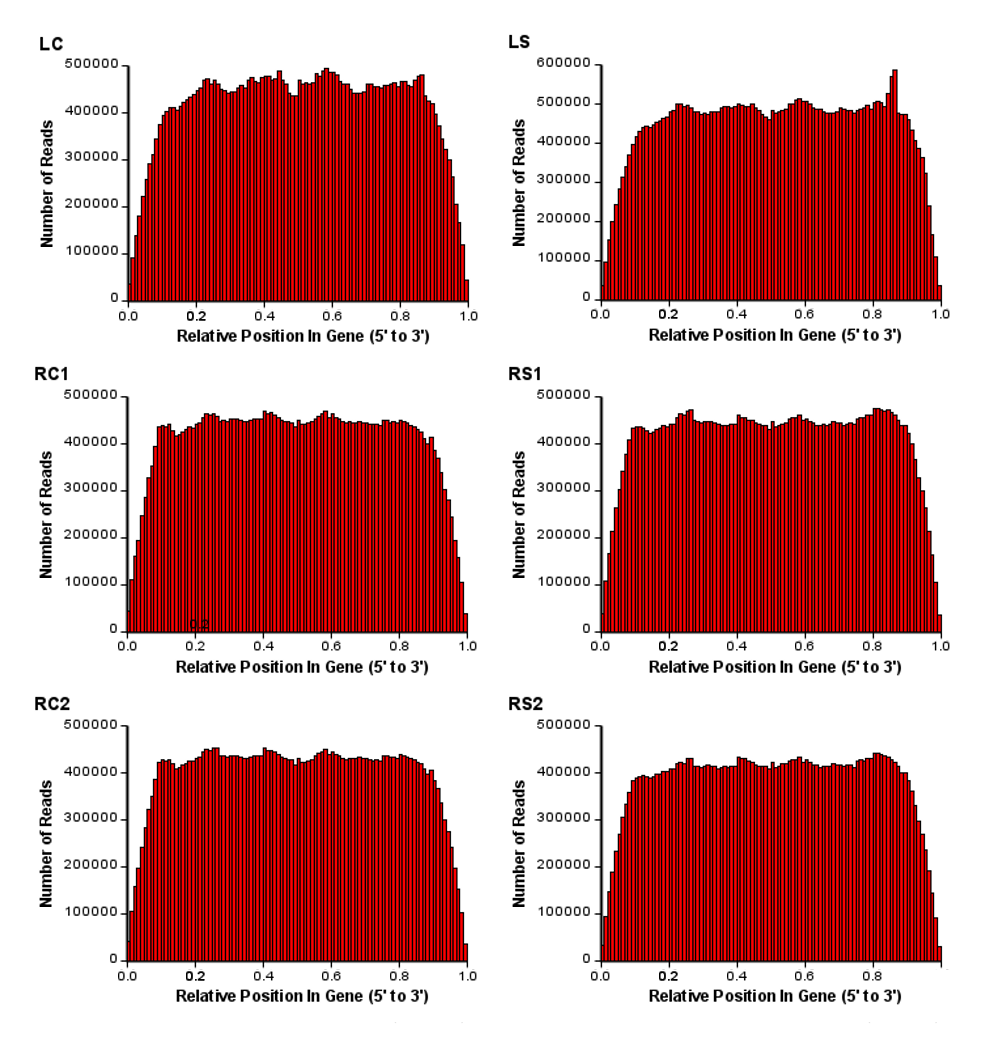

Supplement: Figure S1 — Random distribution of clean reads. The x-axis describes the number of reads and the y-axis indicates the number of clean reads mapped to relative positions in unigenes for the six subtranscriptomes. The orientations of the genes are in 5′ to 3′direction and the gene lengths are normalized. (TIF) [file pone.0092598.s001.tif]

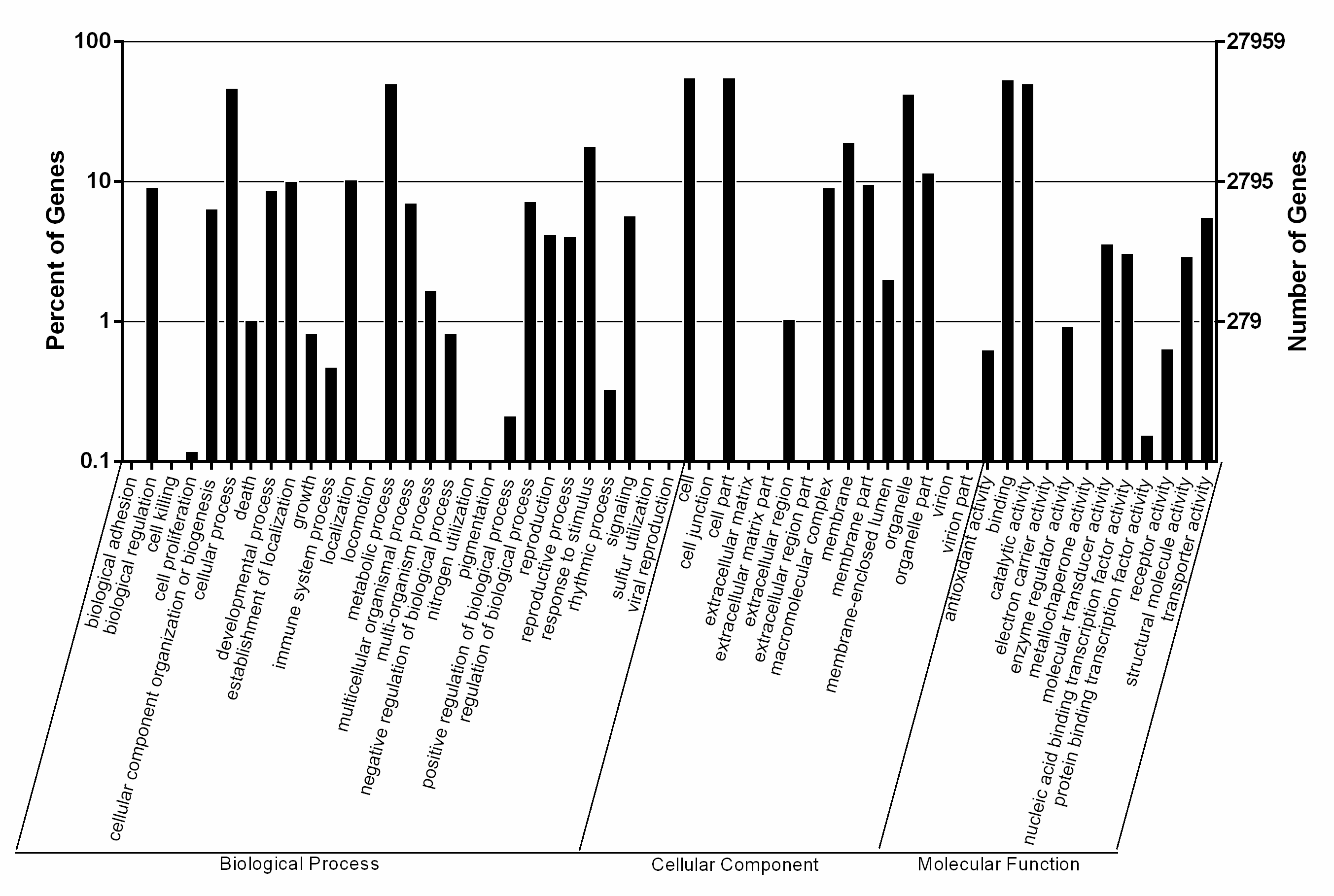

Supplement: Figure S2 — GO annotations of all-unigenes. The annotations were performed with Blast2GO software. The length of each bar indicated the percentage of all unigenes falls under each GO terms. The x-axis is in logarithmic scale. (TIF) [file pone.0092598.s002.tif]

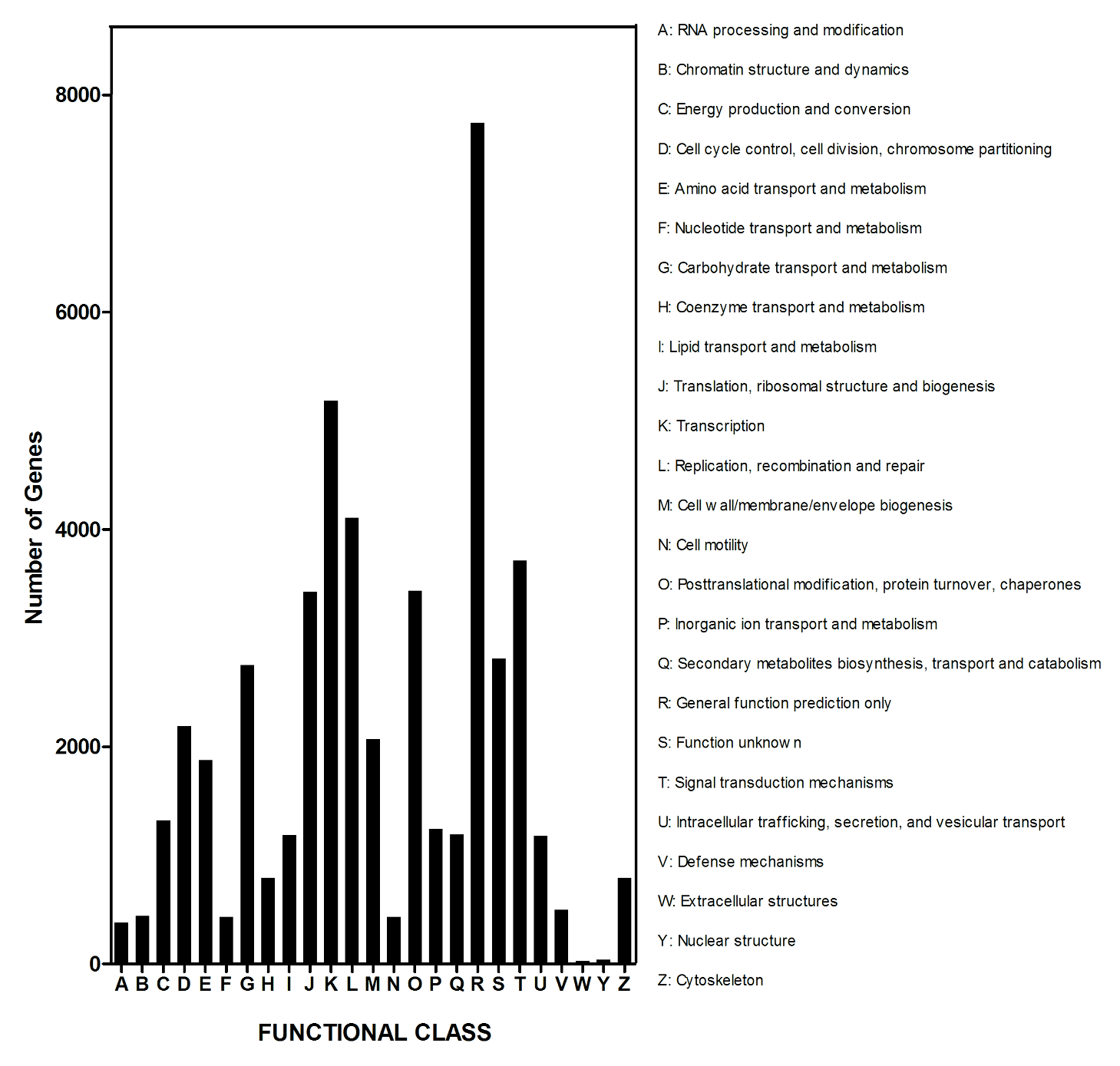

Supplement: Figure S3 — COG function prediction of all-unigenes. The possible functions of all-unigenes were predicted by alignment to COG database. Each letter in the x-axis represented the COG categories listed on the right of the graph. (TIFF) [file pone.0092598.s003.tiff]
